# Endodontic Dentistry: Analysis of Dentinal Stress and Strain Development during Shaping of Curved Root Canals

**DOI:** 10.3390/healthcare11222918

**Published:** 2023-11-07

**Authors:** Laura Iosif, Bogdan Dimitriu, Dan Florin Niţoi, Oana Amza

**Affiliations:** 1 Faculty of Dentistry, University of Medicine and Pharmacy “Carol Davila” Bucharest, 17–21 Calea Plevnei Street, Sector 1, 010221 Bucharest, Romania; laura.iosif@umfcd.ro (L.I.); oana.amza@umfcd.ro (O.A.); 2 Faculty of Industrial Engineering and Robotics, University POLITEHNICA of Bucharest, 313 Splaiul Independenței Street, 060042 Bucharest, Romania

**Keywords:** endodontics, finite element analysis, endodontically treated teeth, dentin, stress, strain, microcrack, dental root canal

## Abstract

Background: Endodontic shaping causes stress and strain in the root canal dentin. Dentin microcracks have the potential to be later followed by root fractures occurring under the occlusal load. The aim of our research was to theoretically determine the values of such dentinal states of stress and strain during the endodontic shaping of curved root canals using finite element analysis (FEA). Methods: To highlight the stress concentrations in dentin, two geometric models were created considering the volume of the curved dental root and the contact between the endodontic file and the root canal walls. The application of forces with different values was simulated both on a uniform curved root canal and on a root canal with an apical third curvature of 25° as they would be applied during the preparation of a root canal. Results: In the case of the first model, which was acted upon with a force of 5 N, the deformations of the root canal appeared along the entire working length, reaching the highest values in the apical third of the root, although there were no geometric changes in the shape of the root canal. Regarding the second root model, with an apical third curvature of 25°, although the applied force was 2 N, the deformations were accompanied by geometric changes in the shape of the root, especially in the upper part of the apical third. At a higher force of 7 N exerted on the endodontic file, the geometric shape changed, and the deformation reached extreme critical values. The resulting tensile stresses appearing in the experimental structure varied similarly to the deformations. Conclusions: Significant stress and strain can develop, especially in the apical third of curved root canals during their shaping, and the risk of cracks is higher for endodontically treated teeth presenting severe curvatures in the apical third of the root.

## 1. Introduction

Endodontic treatment may fail due to various extrinsic and intrinsic microbial and nonmicrobial factors. In the hierarchy of etiologies of endodontic failure, the literature places the persistence of microbial infection in the intra- or periradicular areas first [1]; however, procedural mechanical errors, often affecting inflamed and modified fields, such as the structure of root canal wall dentin due to pulp pathology [2], are also highly responsible for short- or long-term complications in the complex root canal space. The latest generation of scanning microscopy shows irregular and wide structural variation in the dentin structure, especially in the middle and apical regions [3], and these data are carefully considered in the minimally invasive and risk-free shaping concepts of current endodontics [4]. The continuous development of endodontic instruments aims to achieve increased efficiency while minimizing the risks that may occur during root canal shaping in the chemo-mechanical step of the treatment. The introduction, development, and diversification of various rotary nickel–titanium file systems have made it feasible to achieve this goal. These instruments are superelastic and employ certain torque and speed values and different types of rotational motion (constant, reciprocating motion), thus allowing the endodontist to successfully cope with the frequent critical clinical situations involving the unfavorable anatomical configurations of root canals, as in the case of narrower or multiple curved root canals, etc.

Despite the continuous development of new generations of endodontic instruments, with constant improvements concerning the type of rotation, the metallurgical properties of the nickel–titanium alloy used, and the design of the file, stress and strain will inevitably appear in the root dentin, thus leading to the appearance of microcracks. Not infrequently, under the action of excessive occlusal forces, these can evolve into root fractures [5]. Especially when the tensile stress exerted by endodontic instruments on the dentin exceeds the value of the tensile strength of the dentin, the probability of the appearance of dentinal cracks becomes a certainty [6].

These dentin microcracks cause stress concentration areas, which are due to elements that are particularly relevant to the endodontic file used, including an active or inactive tip and the taper, cross-section, pitch, flute, helical angle, cutting angle, and rake angle. The stress concentration on the dentin walls, associated with the risk of cracks at this level, is exacerbated by endodontic instruments with active tips and aggressive cutting contours (which tend to have a screwing effect in the root dentin), those with increased tapering, and those that can cause the accumulation of debris [7]. The removal of root-filling materials can cause additional stress during endodontic retreatment [8], and the type of occlusion and the occlusal load are other variables that can influence the level of dentinal stress [9]. A rotary system consisting of a single engine-driven endodontic file has a higher taper, and although the instrument has periodic clearance at the root canal walls, it can also cause dentin microcracks.

The parameters that define the rotational movement of the instrument, such as speed and torque, are also related to microcracks, and their increase is directly proportional to the number of dentinal microcracks [10].

The internal characteristics of dentin, which presents a hierarchical composite structure, dictate the effects of the stress exerted on the root canal. Other variables must be considered, such as the vitality of the teeth, the time of the endodontic treatment, pre-existing root canal treatments, and the age of the patient. All of this implies different levels of dentine dehydration, which starts with the onset of pulp pathology and increases with the removal of the pulp [11,12]; changes in the mechanical properties of dentin due to the effect of root canal irrigants [13], root canal chemical treatment, and endodontic fillings [14]; and the obstruction of the dentin tubules with age, resulting in a decrease in the effectiveness of mechanisms involved in preserving dentin resistance [15,16,17,18] compared to the capacity of young, opened dentinal tubules to deflect, disperse, and induce the branching of microcracks [19].

So-called dentine defects [20] have a crucial role in the appearance of root microcracks because they facilitate the accumulation of stress during root canal instrumentation [21,22,23]. In addition, the relatively recently determined value of the tensile strength of dentin, about 106 MPa, is three times lower than the stress generated by rotary files on the root dentin [24], which facilitates the development of microcracks. The occlusal forces transmitted through crowns or prosthetic root restorations in the case of teeth with root canal treatments facilitate the development of dentine microcracks even further [25]. The greatest risk of vertical root fracture occurs as the sum of the effects of ductile fractures in the inter-tubular dentin and brittle cracks in the peritubular dentin increases [26], which, in most cases, requires tooth extraction.

The increased interest of endodontists in the in vitro visualization of dentin defects after root canal treatment has thus led, in the last two decades, to the use of numerous complementary methods, such as LED transillumination [20], thermography [27], scanning electron microscopy [28], and many other imaging scans [29,30,31,32], although none of them are reliable in presenting the stress and dentinal deformation developed during root canal instrumentation.

A different approach was therefore required to identify and pinpoint the stresses exerted on the root canal dentin during endodontic instrumentation.

Starting from this objective, the finite element analysis (FEA) technique was initially developed in the 1950s for its application in the aeronautical industry [33] and was used afterward by civil engineers for the numerical analysis of stress distribution and concentration depending on the material properties and loading conditions, thus becoming an extremely feasible technique in determining the behavior of devices or structures under different circumstances [34]. The step from engineering purposes to simulation-based medicine and the development of complex computer models of biological structures was made two decades later, i.e., in the early 1970s [35]. Since then, FEA has been used to evaluate stresses in a variety of fields of clinical medicine, such as cardiology [36] and orthopedic surgery [37,38], as well as numerous specialties of dental medicine, such as oral and maxillofacial radiology [39], oral implantology [40], and restorative dentistry. Restorative dentistry refers to the diagnosis and treatment of diseases of the teeth and their supporting structures for the functional and esthetic purposes of an individual. Restorative dentistry therefore includes various other dental fields, such as prosthodontics, periodontics, and endodontics [41]; in each of these fields, FEA has been and continues to be extensively used.

Especially in the last two decades, FEA has become an important research tool in the field of rotary endodontics [42]. It has made an important contribution to the investigation and evaluation of the clinical performance of various nickel–titanium rotary instruments, such as their superelasticity, toughness, cyclic fatigue resistance, shape memory, torsional strength, etc. The benefits of using FEA for endodontics include the use of discretization for the precise modeling of the complicated geometry of the instruments and of the root canals. The term refers to the subdivision of a continuous structure, such as an endodontic file or a dental root, into simple geometric shapes called elements [43], which are interconnected in their outer nodes and therefore prepared for introduction into the analysis. 

FEA has also been successfully used in endodontics to quantify and analyze the stress distribution introduced into the root canal [44] during its shaping by endodontic files. Using this method, our research aimed to determine and analyze the stress and strain states generated by endodontic instruments on the dentin walls of curved root canals to predict the treatment outcomes in clinical cases showing a high degree of difficulty. More specifically, we evaluated the tensile stress, which is defined as the stress that tends to stretch or lengthen the material (in our case, the root dentin), and the strain, which is measured as the deformation of a solid (also root dentin), during endodontic instrumentation. 

## 2. Materials and Methods

Since the experimental determination of the dentinal stress and strain occurring during endodontic shaping is difficult to achieve, FEA was preferred by the authors to obtain reliable results. Thus, two mathematical models, one of a unique, uniform curved root canal and one of a root canal with an apical third curvature of 25°, were created, and a series of working conditions were simulated. The stress and dentinal deformation that occur during the application of different forces in the long axis of the root were monitored, analyzed, and compared. We considered the following data concerning the rotational movement of the endodontic file: a 360-degree continuous rotational movement at 300 RPM, with a mean torque value of 3 N/cm. Three states of stress were further established, considering the possible forces applied by the clinician on the canal walls when using rotary endodontic files, with values of 5 N, 2 N, and 7 N, respectively. We started from the premise of applying a force at a constant value over the entire working length of the root canal in its coronal, middle, and apical thirds. For the first model, we tested an average force value applied to the endodontic instrument, and for the model with accentuated apical curvature, we used the minimum and maximum possible values, according to the literature for these rotary systems [45,46], generated during the continuous 360-degree rotation at 300 RPM of the endodontic instrument in the root canal. 

The theoretical determination of the states of stress and strain (S) was based upon modeling the process using FEA Tool Multiphysics version 1.10 and ANSYS 19.1 (Inc., Canonsburg, PA, USA), the corresponding software. In terms of contemporary endodontic instrumentation, although they are based on a multitude of rotary systems [47], FEA experimental studies are carried out in a similar way [48].

Knowing the magnitude of the dentinal stress developed during root canal shaping is necessary because when its value exceeds the tensile strength, i.e., the elastic modulus (Table 1), dentinal microcracks and eventually even cracks will develop, as previously demonstrated [49].

**Figure 1:**
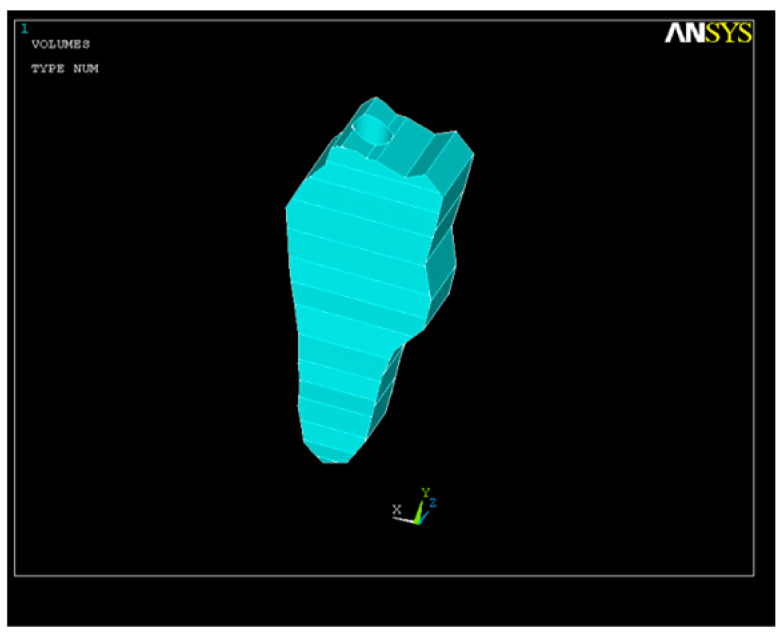
Dental volume geometry.

**Figure 2:**
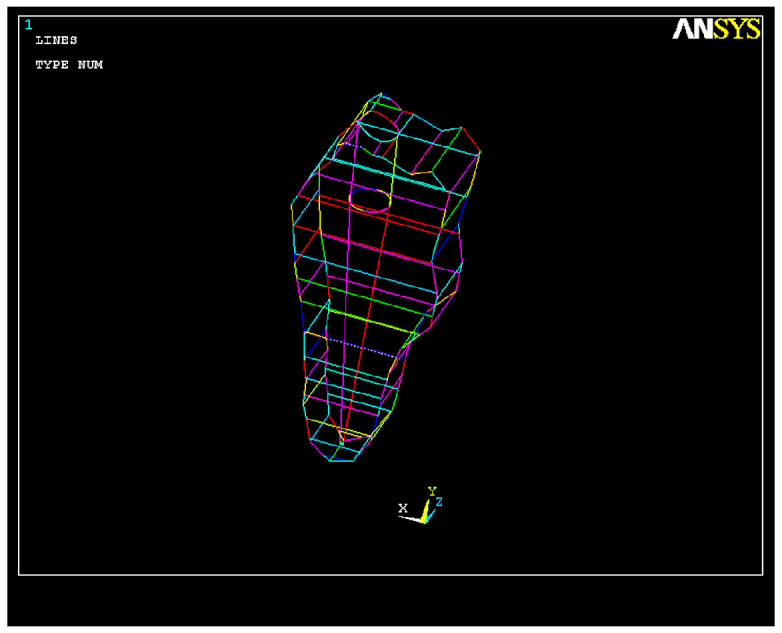
Root canal geometry.

In our study, we first used FEA to simulate the volumetric (Figure 1) and geometric characteristics (Figure 2) of the whole tooth and of the root canal, respectively.

The desired geometric models were then created and discretized, and the working conditions were introduced and exported for FEA. Thus, the following work sequences were completed.

### 2.1. Discretization of the Tooth Structure and Root Canal

**Figure 3:**
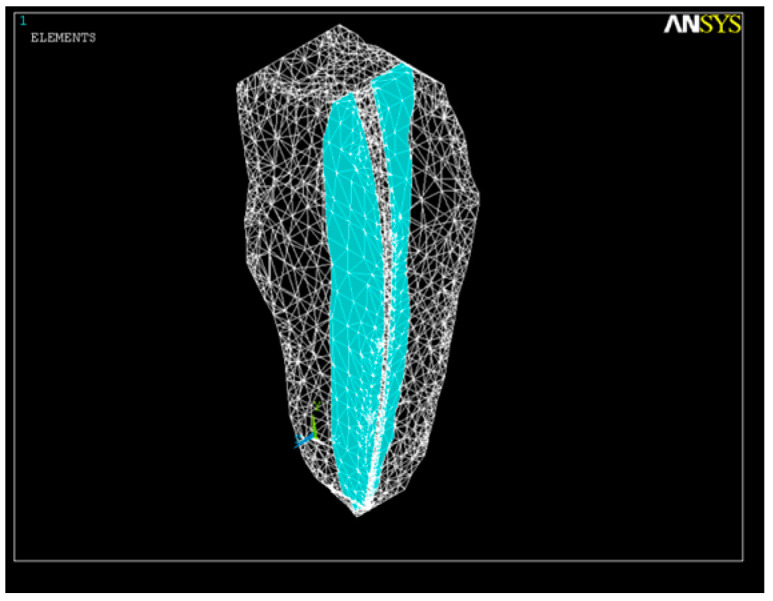
Discretization of the tooth structure and root canal.

According to various Cone-Beam Computed and Micro-Computed Tomography studies [54,55], we considered the root canal cross-section to be a spline curve (Figure 3) with the ideal diameter of the apical constriction Φ_c_ = 0.15 mm [56]. The forces exerted by the multiple endodontic rotary files used during root canal shaping act evenly upon the root canal dentin.

**Figure 4:**
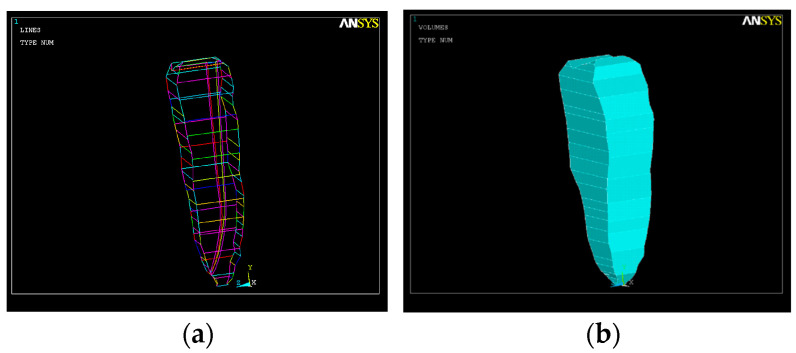
The root canal with an apical third curvature of 25°. (**a**) Volumetric image; (**b**) image of the root canal.

The second simulation used a model considering an apical root canal curvature of 25° with respect to the long axis with the volumetric geometry shown in Figure 4.

### 2.2. Establishing the Working Premises

**Figure 5:**
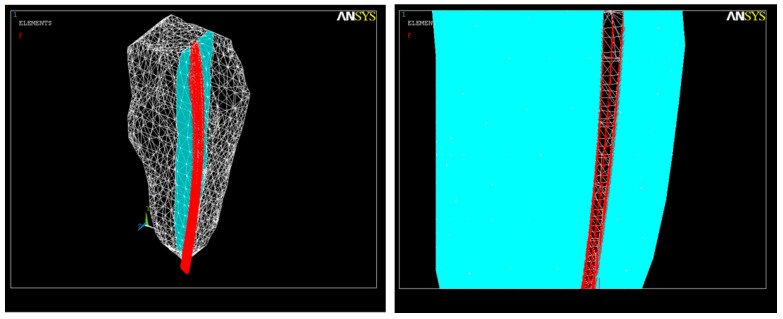
Application of forces during the root canal preparation.

In this study, the contact between the endodontic instrument and the root canal dentin was ideally considered to be continuous on the whole circumference of its cross-section, thus allowing the existence of forces that act uniformly on the dentinal walls along the entire length of the root canal (Figure 5).

### 2.3. The Embedding of the Dental Root into the Bone Structure

**Figure 6:**
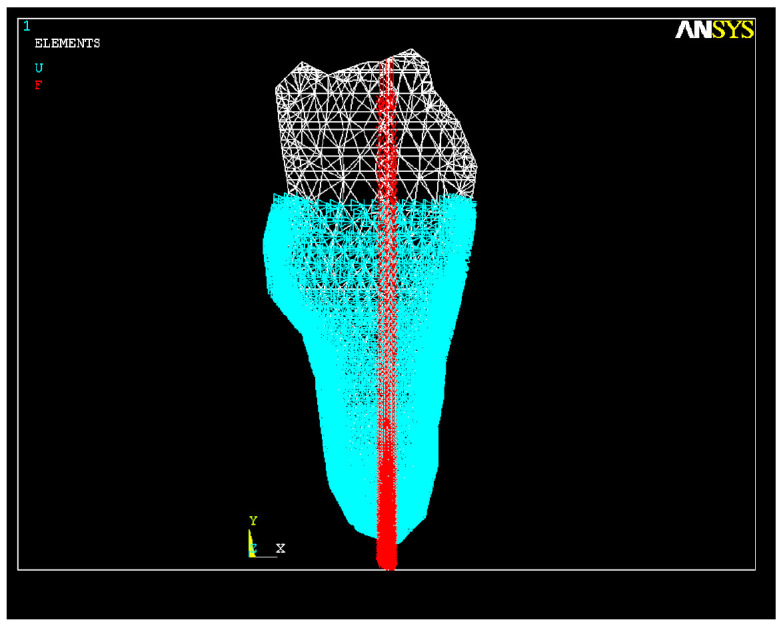
Embedding the dental root into the bone structure.

The graphic image of this implantation in the alveolar bone structure was made as previously mentioned, namely, for a single-rooted tooth with a curved canal, as shown in Figure 6. 

## 3. Results

### 3.1. Dentinal Stress and Strain Occurring during Endodontic Instrumentation of a Curved Root Canal

**Figure 7:**
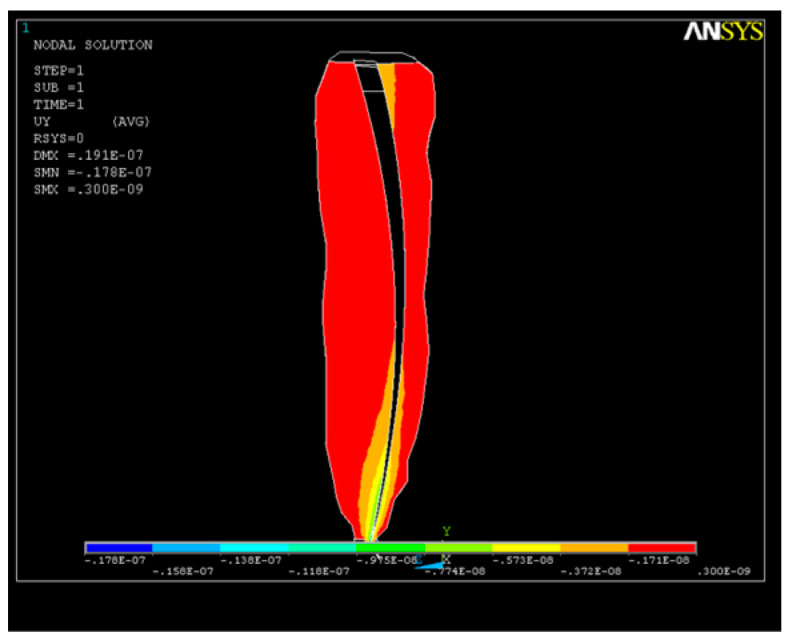
Dental root structure strains on OY axis in the case of application of a force F _tot_ = 5 N.

The analysis performed mainly consisted of determining the strain and the state of stress that appeared in the studied structure by simulating the application of forces with different values during the shaping of a curved root canal. In the case of applying a force of 5 N, structural deformations were evident, with higher values in the apical third of the root (Figure 7).

**Figure 8:**
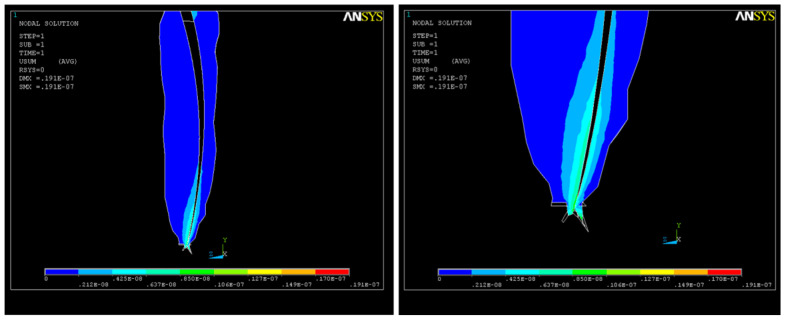
The sum of the deformations in the case of application of a force F _tot_ = 5 N.

The deformations in this case are presented in Figure 8.

**Figure 9:**
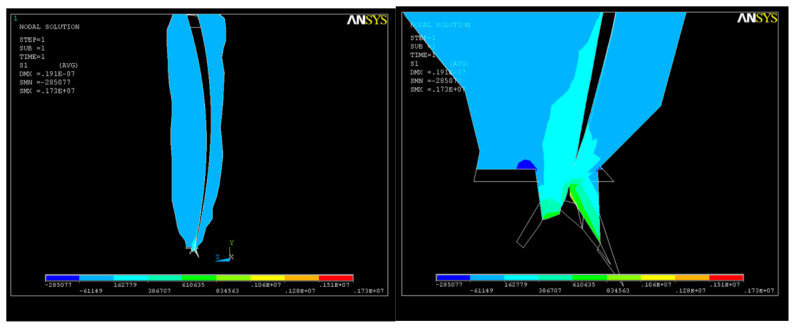
S_1_ stress due to the action of endodontic instruments upon the apical third of the root canal.

The consequence of the action of this system of forces was the appearance of an S_1_-type tension state in the apical third of the root canal (Figure 9), with a lower value than that corresponding to the elastic modulus of dentin. The maximum value of the tensile stress in the curved area was σ_imax_ = 0.09 × 10^8^ N/m^2^. This state of tension, which mainly assesses the tensile stresses, even if it does not produce geometric changes in the root canal, generates stresses in the dentin, which must be considered.

**Figure 10:**
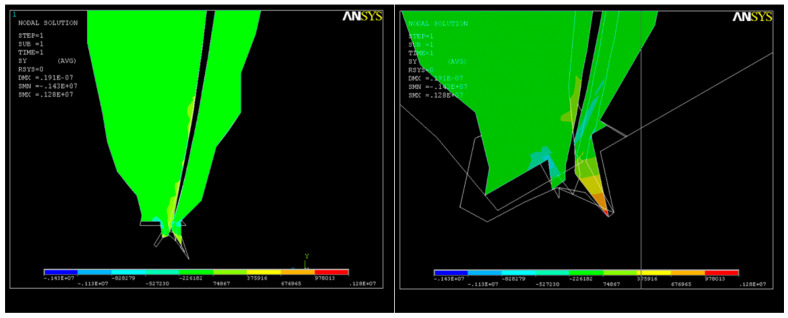
Axial section of the dental structure along the OY axis—stress in the apical third.

**Figure 11:**
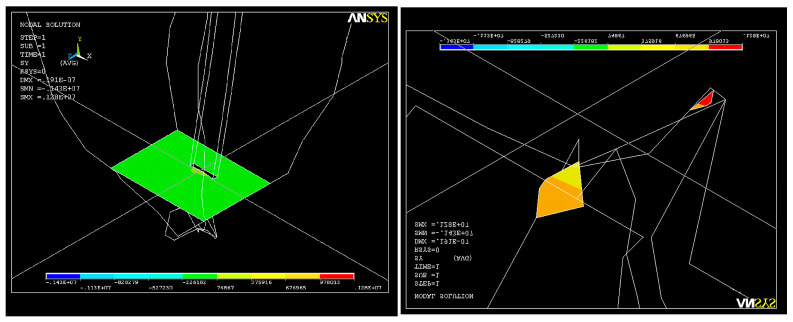
Cross-section of the dental structure for the OY axis—stress in the apical third.

The state of stress along the OY axis is shown in Figure 10; their values are close, with the maximum also being in the apical third, where the volume of the root canal is smaller and the risk of cracking is higher. As a result of the action of the system of forces, a state of deformation (strain) appears in the structure, with higher values in the apical third area that can reach up to UY_max_ = 0.05 × 10^−4^. For better visualization of the state of stress and strain that occurs, further sections were made in the areas depicted in Figure 10 and Figure 11. 

### 3.2. Dentinal Stress and Strain Occurring during Endodontic Instrumentation of a Root Canal with an Apical Third Curvature of 25°

**Figure 12:**
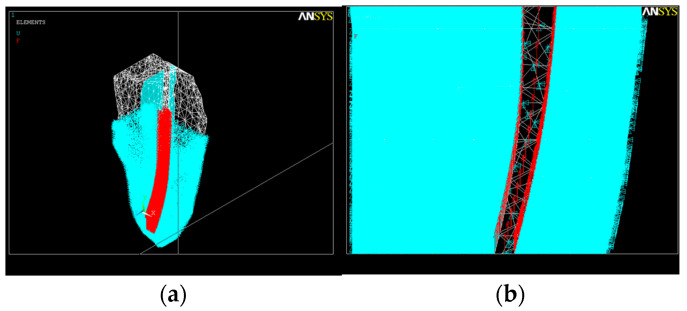
Force distribution inside the root canal with a 25° curvature. (**a**) Discretization and force distribution in nodes; (**b**) section in the apical third.

After the discretization was complete, it was assumed that the acting forces were axially distributed in each node of the network, with the nodes being unevenly distributed by the software itself (Figure 12). A force of 14 N acts on each node if a force of 2 N is applied to the endodontic instrument.

**Figure 13:**
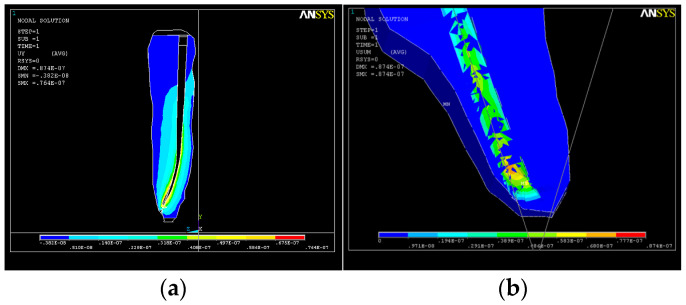
Structural deformation: (**a**) deformation of the dental root along the OY axis; (**b**) deformations in the apical third.

As a result of the action of the force system, a state of deformation (strain) appears in the structure (Figure 13a), with much higher values in the apical third (Figure 13b) that can reach up to UY_max_ = 0.08 × 10^−4^.

**Figure 14:**
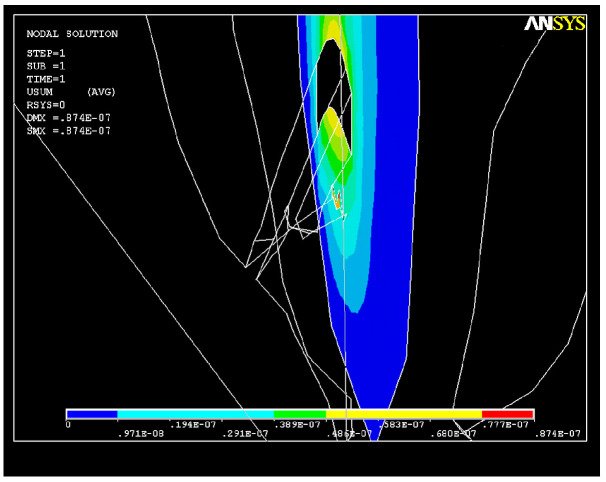
Long axis showing the structural deformation.

A long-axis section through the dental root showing the deformations is depicted in Figure 14. Due to the action of the endodontic instrument, changes in the shape in the upper part of the apical third of the root canal can be noticed.

**Figure 15:**
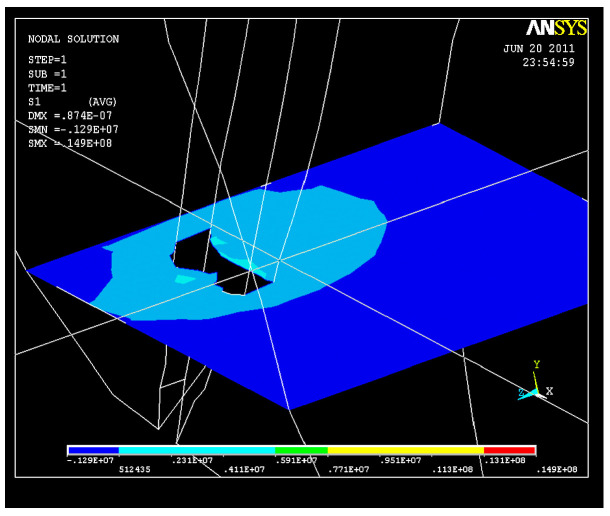
S_1_ stress map in the curved area: σ_imax_ = 0.14 × 10^8^ N/m^2^.

Following the action of the force system on the structure, a state of tension also appears, with the maximum value of the tensile stresses in the curved area being σ_imax_ = 0.14 × 10^8^ N/m^2^ (Figure 15), a value that cannot be neglected, though not exceeding the breaking strength corresponding to the elastic modulus of dentin. 

**Figure 16:**
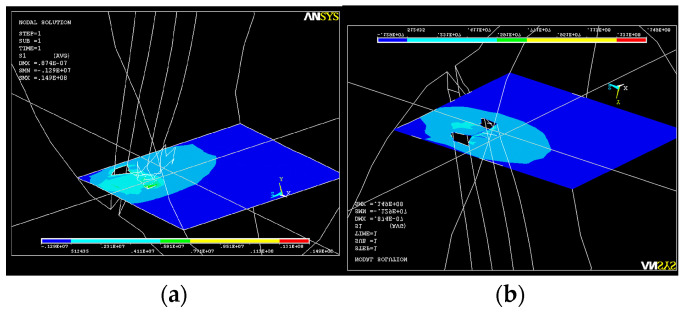
S_1_ stress map in a cross-section of the root canal: (**a**) in the middle of the apical third; (**b**) at the end of the apical third.

Two consecutive cross-sections of the apical third of the root canal show how the action of the system of forces increases S_1_ tensions in this area (Figure 16).

**Figure 17:**
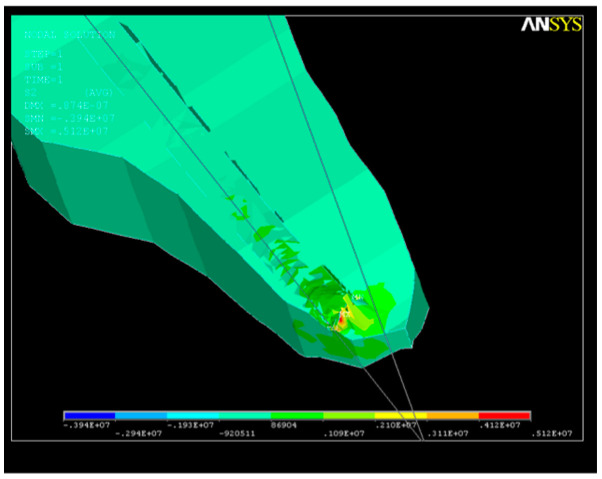
State of tension of type S_2_ at the tip of the apical third of the root canal.

The state of tension of type S_2_ at the end of the apical third shows the same tendency toward the narrowing of the root canal (Figure 17).

**Figure 18:**
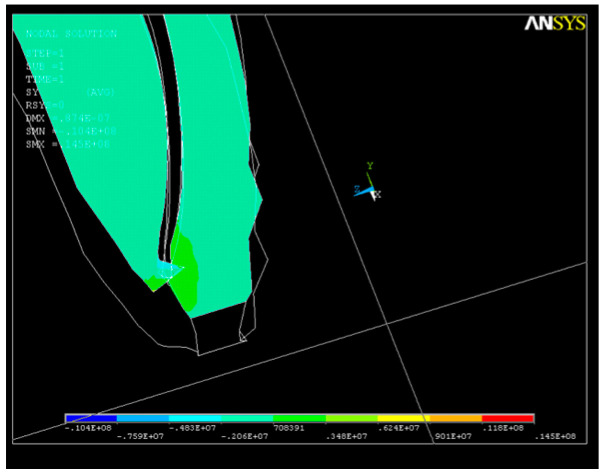
Stress map in the OY direction in the apical zone.

Tensions on the OY axis at the apical constriction clearly show the end of the root canal (Figure 18). The need to relieve the stress to avoid the development of microcracks over time and, eventually, cracks in the dentine becomes obvious in this situation.

**Figure 19:**
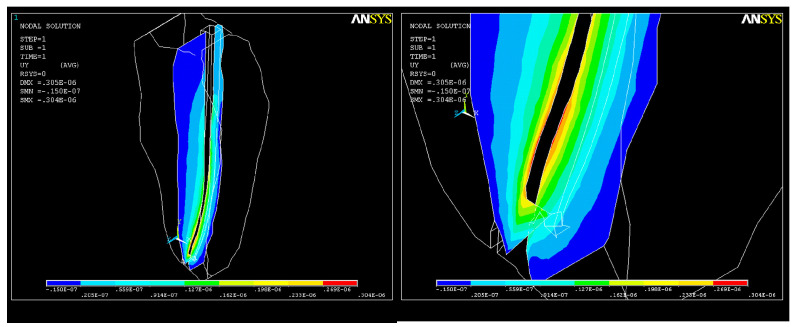
Deformations along the root canal in the case of total loading F _tot_ = 54 N; UY_max_ = 0.3 × 10^−4^.

If a force with a value of 7 N is exerted on the endodontic file, each node of the network will show a total resulting force F _tot_ = 54 N (Figure 19). This value is an unusual one and leads to the deformation of the structure up to a maximum value of UY_max_ = 0.3 × 10^−4^.

**Figure 20:**
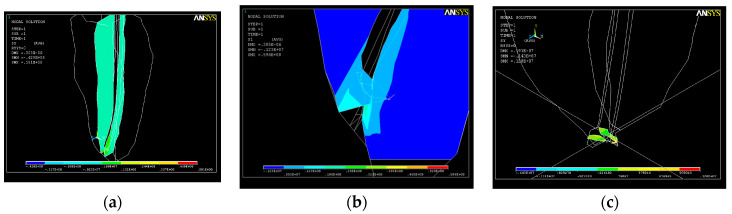
Stress map in the root canal: (**a**) SY stress along the curvature; (**b**) SY stress in the apical third; (**c**) SY stress at the apex.

The state of stress that appears under the action of the system of forces on the dentin–endodontic instrument interface is shown in Figure 20. The magnitudes of the main S_1_ stresses in the apical third area, with a maximum value of tensile stress of σ_imax_ = 0.49 × 10^8^ N/m^2^, are similar to those of SY stresses at the curvature level, almost completely covering the end of the root canal.

For a better visualization, all of these results are included in Table 2.

## 4. Discussion

Root canal curvatures are present in most teeth [57], including anterior but mainly posterior ones, always posing a major challenge to clinicians during endodontic treatment, regardless of the shaping technique being used. The most common iatrogenic endodontic complications, such as blockages, lacerations, and root canal perforations [58], are usually observed with the use of improper instrumentation techniques, especially through the inaccurate handling of endodontic instruments [57] and the application of excessive forces. 

In this regard, the axial and rotational movements of the endodontic instrument in the root canal have the most important impact on the apical third because the largest values of friction with the dentin walls occur at this level. Maximum stress and strain will develop due to cyclic bending and thus implicit alternating elongations and compressions of the endodontic instrument, depending on its direction of rotation and position with respect to the root canal curvature, and also due to the forces exerted on the endodontic instrument. The tensions occurring in the dentinal structure during root canal shaping are therefore necessary to consider because their overlapping with those due to the occlusal load can possibly overcome the breaking strength of the tooth structure and thus lead to cracks or even root fractures.

The root canal geometry is particularly complex, displaying curvatures along all three axes [59]. The root canal curvatures can be further differentiated based on the angle of curvature, such as straight (5° or less), moderate (10–20°), and severe (25–70°) [60]. Each root canal therefore has unique particularities concerning the shape and dimensions; from this point of view, designing a valid universal model appears to be virtually impossible. During root canal instrumentation, the action of the endodontic instrument is especially exercised in the curvature area in an uneven way, with a tendency to block its tip in the apical third and leave the canal unshaped in this area. The action of shaping is amplified on the inner curvature of the root canal.

In our FEA study, the working conditions consisted of the application of different forces on the endodontic instruments developed at the interface between the endodontic file and the root canal walls, preceded by the mathematical determination of the tooth’s embedment area in the bone structure. We considered stress and strain distributions during the use of endodontic instrumentation in two selected root canal models: one simulating a single uniformly curved root canal and another single curved root canal with an apical third curvature of 25°. In the case of the first experimental model, when applying a force of 5 N, the root canal deformations appeared along the entire working length, reaching the highest values in the apical third of the root. These values did not, however, exceed the elastic modulus of dentin and thus were not accompanied by any geometric changes in the shape of the root canal. Regarding the second root model, with an apical third curvature of 25°, although the applied force was less than half of the first one, i.e., only 2 N, the deformations were accompanied by geometric changes in the dental root, especially in the upper part of its apical third. With a higher force of 7 N exerted on the endodontic instrument, the geometric shape changes, and the deformation reaches critical values, larger than the cross-section of the root canal in the apical third, thus leading to the impossibility of shaping the root canal in this area. It must be underlined that, in all cases with curved canals simulated by us in the present study, the highest stress and strain values were distributed in the apical root third, which is also in accordance with studies conducted on teeth with straight root canals [61]. Although extensive FEA experimental studies have been performed in endodontics, with one of the main objectives being the identification of the forces generated during root canal shaping, comparing our results to the values obtained by other authors was somewhat difficult, because experimental root canal models remain unstandardized [42]. The valuable findings of Lee et al. [62] and Basheer Ahamed et al. [63], who also described different root canal geometries but did not disclose the origin of or rationale for their chosen dimensions and parameters, converge with our observations and showed that the increased curvature of the root canal generated higher stresses.

Moreover, the state of stress and deformation is totally different depending on the angulation of the root area where the analysis is performed and on the system of forces acting on the endodontic instrument used to shape the root canal. According to our study, even when low forces were applied on the endodontic instruments, the greater the curvature of the root canal, the greater the risk of geometric changes in the shape of the root canal in the apical third. However, our results do not overlap with the earlier findings of Versluis A. et al., who compared the distribution of the stress developed during root canal preparation depending on its round or oval cross-section. Round canals exhibited lower uniform distributions, whilst oval canals showed uneven distributions with high concentrations at the buccal and lingual canal extensions and greater stresses in the coronal and middle thirds than in the apical one [64]. In contrast, all endodontic instruments based on conventional or heat-treated alloys tested by Prati C. et al. [65], regardless of their geometrical features (cross-section, taper) or the setting of the modulus of elasticity of the dentin (18 and 42 GPa), generated a stress area concentration corresponding to the root canal curvature at approx. 7 mm from the apex, which is in the coronal portion of the apical third, similar to our findings.

At this point, some limitations regarding our experimental results need to be acknowledged and highlighted. One aspect refers to the fact that the rotary system we investigated only uses conventional continuous rotational movement. In recent years, rotary files with reciprocating motion have also been used in endodontic treatment, and there are studies showing their greater accuracy in achieving apical-third shaping, even in cases of more substantial canal curvature [66]. Thus, a comparison between the two types of rotation motions in terms of stress and strain development under the same simulated conditions could have provided even more representative data for clinicians. Another shortcoming of our study consisted of the representation of a root canal configuration model with a uniform dentine density. The role of irregular surfaces in the canal configuration, the presence of predentin or dehydrated dentine, and different levels of dentine hardness, which may create differences in the stress distribution along the working length of the root canal, were not considered. 

Our simulation considered strictly 360-degree continuous rotation motion at 300 RPM, with a mean torque value of 3 N/cm. In clinical reality, the motion of endodontic instruments is a combined one, consisting of the simultaneous rotation and axial withdrawal of the file from the root canal. This vertical component generates additional stress and strain on the dentin walls. This is another limitation of our study and a starting point for further research. The alveolar bone, with characteristics related to the type and density, and the periodontal ligament were also not simulated in our experimental research. In this regard, Rathi A. et al. [67] proved the hypothesis that the periodontal ligament may also intervene in stress distribution during endodontic shaping.

Although the distribution of stresses will vary amongst teeth according to the individual root shape and root curvatures, the specific duration of force application during endodontic shaping, and possibly other parameters, such as tooth age and occlusal load, the principles of changes in stress distribution reported here should be considered in actual clinical situations. FEA enables an explicit picture of what happens inside the curved root canal during its endodontic instrumentation and may provide a useful behavioral “pattern”. There is currently only one other tool that provides in vivo insight during the shaping of the root canal, namely, the apex finder, which reveals the axial level reached during instrumentation in relation to the apical constriction [68]. There are no practical means to provide information about the dentinal states of stress and strain developing during endodontic treatment, thus making finite element analysis a valuable aid for researchers and opening up new clinical perspectives concerning the conservative shaping of root canals with critical curvatures. 

## 5. Conclusions

Currently, computational methods come to the aid of most fields of medical research, making it possible to provide information when conventional methods are hard to be successfully applied. In the experimental conditions of our investigation in such a difficult clinical working space, represented by the root canal, it was proven that FEA provides very precise and predictable data regarding the consequences of endodontic instrumentation on root canal dentin, thus contributing to a reduction in the immediate risks and long-term complications of endodontic treatments.

Our FEA study showed that significant stress and strain can develop, especially in the apical third of curved root canals during their shaping, regardless of the type of rotary instrument used. These values, in turn, depend on the force applied to the endodontic instrument. In this regard, cracks and, ultimately, fractures in the dental structure may develop when exceeding a certain threshold. The risk is higher for teeth presenting severe curvatures in the apical third of the root. 

## Figures and Tables

**Table 1:** Mechanical properties of dentin.

Properties	Value
Elastic modulus—Young’s modulus	19.794 ± 0.93 GPa [50]
Hardness	0.65 ± 0.52 GPa [50]
Density	2.12 ± 0.1874 g/cm^3^ [51]
Poisson’s ratio	0.29–0.31 [52,53]

**Table 2:** Stress and strain developed in the investigated root canal models.

Root Model	Force Applied	Stress (Tensile Stress)	Strain (Deformation)
1. Root canal with uniform curvature	5 N	0.09 × 10^8^ N/m^2^	0.05 × 10^−4^
2. Root canal with 25° curvature in the apical third	2 N	0.14 × 10^8^ N/m^2^	0.08 × 10^−4^
	7 N	0.49 × 10^8^ N/m^2^	0.3 × 10^−4^

## Data Availability

The data presented in this study are available from the corresponding authors upon reasonable request.
